# Supplementary material for: HIVprotI: an integrated web based platform for prediction and design of HIV proteins inhibitors
Source: J Cheminform. 2018 Mar 9;10:12. doi: 10.1186/s13321-018-0266-y (PMC5845081; doi:10.1186/s13321-018-0266-y)
Supplement: Supplementary file 2 — Additional file 2. Source code of HIVProtI web server. [file 13321_2018_266_MOESM2_ESM.zip › HIVprotI_Source-code/hivprotI/weka-3-6-6/doc/weka/gui/streams/package-frame.html]

weka.gui.streams


weka.gui.streams

Interfaces 
  
*InstanceListener*
  
*InstanceProducer*
  
*SerialInstanceListener*

Classes 
  
InstanceCounter
  
InstanceEvent
  
InstanceJoiner
  
InstanceLoader
  
InstanceSavePanel
  
InstanceTable
  
InstanceViewer
  
Messages
